# Judgment of togetherness in performances by musical duos

**DOI:** 10.3389/fpsyg.2022.997752

**Published:** 2022-11-18

**Authors:** Sara D'Amario, Werner Goebl, Laura Bishop

**Affiliations:** ^1^Department of Music Acoustics, Wiener Klangstil (IWK), mdw – University of Music and Performing Arts Vienna, Vienna, Austria; ^2^RITMO Centre for Interdisciplinary Studies in Rhythm, Time and Motion, University of Oslo, Oslo, Norway; ^3^Department of Musicology, University of Oslo, Oslo, Norway

**Keywords:** togetherness, ensemble performance, motion capture, joint action, music perception, flow, interpersonal synchronization

## Abstract

Musicians experience varying degrees of togetherness with their co-performers when playing in ensembles. However, little is known about how togetherness is experienced by audiences and how interpersonal dynamics in body motion and sound support the judgment of togetherness. This research investigates audience sensitivity to audio and visual markers of interperformer coordination and expressivity in ensembles, in relation to modality of stimulus presentation and audience music background. A set of duo ensemble performances, comprising motion capture recordings of the musicians' upper bodies and instruments, were presented to participants with varying music background, including novices and semi-professional musicians. Participants were required to: (i) watch and listen, (ii) only watch, and (iii) only listen to the selected recordings, whilst providing dynamic ratings of how much togetherness between musicians they perceived. Results demonstrate that sound intensity and similarity in right arm motion (quantified using cross-wavelet transform analysis) were significant predictors of rated togetherness in novices, whilst sound synchronization and chest motion coordination predicted togetherness responses in semi-professional musicians. These results suggest the relevance of the quality of body motion coordination and of certain features of the audio outputs in the audience perception of togetherness. This research contributes to a better understanding of the perceptual mechanisms supporting socio-cognitive judgments of joint action activities.

## 1. Introduction

During music ensemble performances, musicians experience varying degrees of musical, cognitive and emotional alignment with their co-performers, that is, varying intensities of musical togetherness (Sawyer, 2006; Seddon and Biasutti, 2009; Hart et al., 2014; Hart and Di Blasi, 2015; Gaggioli et al., 2017). Togetherness experiences are socially and aesthetically rewarding (Berliner, 1994), and can change in intensity over time as the quality of interactions between group members evolve. Aspects of togetherness have been investigated through studies of synchronization in body motion (Hart et al., 2014) and note timing (Wing et al., 2014). Strong experiences of togetherness may be associated with shared absorption or group flow, colloquially described as being “in the zone” (Sawyer, 2006; Gaggioli et al., 2017), and characterized by seemingly-effortless collaboration between group members as well as behavioral and physiological synchrony (Hart et al., 2014). Playing music with others, or simply synchronizing rhythmic body movements, can strengthen the relationships between musical partners more broadly, with effects that persist beyond the end of the performance. Prosocial benefits of rhythmic synchronization have been observed for infants (Cirelli et al., 2014a,b), pre-schoolers (Rabinowitch and Meltzoff, 2017a,b), and adults (Valdesolo et al., 2010; Mogan et al., 2017).

A growing corpus of research has focused on experiences of togetherness that are self-reported by performers in amateur musical bands (Gaggioli et al., 2017), experienced musical and improvising groups (Hart et al., 2014; Hart and Di Blasi, 2015), professional ensembles (Seddon and Biasutti, 2009), and therapeutic settings (Smetana et al., 2022). However, very little is known about how audiences judge togetherness and how togetherness manifests in musicians' body motion and musical outputs. This study investigates how an audience evaluates the strength of togetherness between musicians during piano duo and clarinet duo performances, and how this judgment relates to specific visual and audio features of the performance. This research contributes to a better understanding of how people communicate the quality of their social interactions through non-verbal behavior (see also Aucouturier and Canonne, 2017; Lee et al., 2020).

The following sections provide an overview of how auditory and visual information shape audiences' experiences of performed music and how music expertise changes audience perceptions of ensemble coordination.

### 1.1. Evaluation of music performance based on visual cues

Musicians' body motion is a core element that can influence listeners' experiences of music (Leman et al., 2017). In addition to some aspects of motion supporting sound production and modifying the sound, certain aspects of body motion (referred to as “ancillary motion”) facilitate coordination and interaction between co-performers (Jensenius et al., 2010) and can also influence listeners' experience of the music that they see performed (Jensenius et al., 2010; Leman et al., 2017). Head movements can relate to the emotionally expressive intentions that musicians aim to communicate. The amount of information flow (measured in the head motion) between members of a professional trio was found to be higher when playing with emotional expression rather than performing mechanically without expression (Chang et al., 2019).

Ancillary motion can also be shaped by the ensemble context and become more predictable in certain situations. Distinctive patterns in the head movements of first violinists differentiate solo and ensemble performances, with head movements being more predictable when the first violinists performed with a string quartet than solo. Visual signals can also reflect the leader-follower relationships between co-performers. Assigned leaders in string quartets tend to influence others more than they are influenced by others, as can be seen in their head motion (Chang et al., 2017). Assigned leaders in piano duos tend to raise their fingers more than assigned followers (Goebl and Palmer, 2009).

Head movements also contribute to the synchronization of certain parts of the music. Certain acceleration patterns in head gestures (i.e., instances of deceleration following acceleration peaks) facilitate piece entrances in piano duos by communicating beat position (Bishop and Goebl, 2017). Increased quantity of head movements in irregularly-timed passages compared to other parts of a piece ease interpersonal coordination during these periods of temporal instability (Bishop et al., 2019b). In summary, musicians' body motion is tied to individual and group expressivity, becomes more predictable in ensemble settings, and can facilitate coordination between ensemble members.

A line of research on audience perception has investigated how audiences perceive musicians' body motion, and have shown that audience members can distinguish the expressive content of a music performance based on musicians' body motion. Members of an audience rating silent video clips were able to detect happiness, sadness and anger in clips where musicians intended to communicate these emotions to others (Dahl and Friberg, 2007). Anger was mostly communicated through jerky movements, happiness through large movements, and sadness through slow and smooth movements. Audio and visual cues interact when the music is both heard and seen, such that happiness and sadness are perceived more accurately when accompanied by compatible music (e.g., happy music accompanying happy interactions) than incompatible music (e.g., happy music accompanying sad interactions) (Kaiser and Keller, 2011). Audiences can also distinguish between reduced and exaggerated levels of expressive intensity in performances by pianists (Vuoskoski et al., 2014) and clarinetists (Vines et al., 2011). The kinematic features of conductors' gestures also inform the perception of expression, and higher ratings of expressivity in conductors' gestures were found to be correlated to higher amplitude, variance, and speed of movement of the conductors (Luck et al., 2010).

In addition to the expressive content, listeners can also gain information about the social interactions between musicians based on their body motion. Regardless of their music background, listeners can distinguish different social intentions (i.e., dominance, insolence, caring, conciliatory, and disdainful) conveyed between musicians in video and sound recordings of jazz duo performances (Aucouturier and Canonne, 2017). Trained musicians can detect leadership dynamics between members of a conductor-led violin ensemble (D'Ausilio et al., 2012), as it was found that an increased influence of the conductor on the musicians related to improved ratings of performance quality. Furthermore, listeners can detect social bonds in group dance (Lee et al., 2020); specifically, ratings of formidability and social closeness were found to be higher in unison rather than coordinated dancing, implying that, in order for perception of social bonds to be maximized, movements should be fully synchronized. In summary, audience members gain information about the expressive content of music and the relationships between musicians by watching musicians' body motion. However, it remains unclear what body motion communicates about musicians' experiences of cohesion. This study aims to address this question with an investigation of how different body motion features contribute to listeners' evaluation of the degree of togetherness between musicians during ensemble performances.

The coupling in periodic body motion that arises between ensemble co-performers may have a particular effect on how audiences judge ensemble coordination. Eerola et al. (2018) observed that over 80% of interaction bouts in non-pulsed, free duo improvisations, manually annotated by experts, were predicted by strength of body movements coordination in common periodicities. In a follow-up study, Jakubowski et al. (2020) analyzed the perception of interpersonal synchrony in improvised duo performances, and found that ratings of synchrony were positively related to measures of common periodic movements of the two performers. Researchers in these studies used computer vision techniques to measure the coordination between musicians related to the overall body motion. However, it remains unclear whether judgments of interactions between musicians might depend on the coordination strength of specific body parts. Head motion, closely tied to visual expressivity (Goebl and Palmer, 2009; Keller and Appel, 2010; Glowinski et al., 2013; Leman et al., 2017; Bishop et al., 2019b) might be more relevant to perception of coordination than other body parts such as hand motion, which is highly dependent on the technical demands of sound production. The current study analyzes audience perception of coupling in periodic motion of musicians' heads, chests, shoulders and arms. This study achieved this through motion capture data analysis, aiming to identify the individual contribution of multiple body parts of interest.

### 1.2. Evaluation of music performance based on auditory cues

Audiences are sensitive to changes in many audio features in music. Differences in loudness, a parameter informative of musical expression, to some extent, can be distinguished regardless of the listeners' training and familiarity with the music being listened to. Manipulations in acoustic intensity induce changes in listeners' perception of the levels of arousal expressed in music (Dean et al., 2011). Audiences relate loudness to musical expression and emotional arousal (Dean et al., 2011) with soft music rated as more pleasant and less energetic and tense than loud music (Ilie and Thompson, 2006). Acoustic intensity also relates to perceived effort or force (Olsen and Dean, 2016).

Furthermore, audiences are, to some degree, sensitive to synchronization between musicians in ensemble playing. It has been reported that listeners without specialized music training were sensitive to the variability of note onset asynchrony and degree of correction gain (i.e., the size of the adjustments relative to the asynchrony), when judging the level of togetherness in computer-simulated string quartet performances of a short excerpt from Haydn's String Quartet Op. 74 No. 1 (Wing et al., 2014). More recently, it has been clarified that listeners, regardless of their music training, can perceive differences in asynchronies in singing ensembles only above a certain threshold placed somewhere between 10 and 38ms. In a study involving singing duo tokens and singing quintet performances, it has been shown that listeners were able to distinguish asynchronies in duo performances that differed on average by 38 ms, but were not able to perceive differences between singing quintet performances that differed in synchrony by only 10ms on average (D'Amario et al., 2019). The preference of the degree of synchronization was also investigated in a set of jazz trio performances comprising the original performances (with asynchronies up to 26ms) as well as recordings manipulated with increased and reduced asynchronies. Results suggest that listeners, regardless of their music training, preferred ensemble performances containing asynchronies as accurate as in the original recordings or even smaller than 19ms but with natural temporal variabilities rather than performances with increased asynchronies (Hofmann et al., 2017).

In summary, audiences can perceive changes in the acoustic intensity of the music performances and in the interpersonal synchronization between musicians. These two parameters, contributing to the expressive content of the performance and boosting performance excellence, may represent auditory cues to the perception of feelings of togetherness. This research tests this hypothesis, by analyzing whether audio features of the music performances such as sound intensity and synchronization predict togetherness ratings.

### 1.3. Multimodal evaluation of music performance

Human judgements of music performances are formed based on both visual and auditory information, if both modalities are available. However, listeners are unreliable in their ability to pair audio and visual cues, as shown by studies observing that participants gave different expressivity ratings to the same audio performance when it was paired with different videos (Behne and Wollner, 2011). A growing body of empirical research has demonstrated that a number of different structural and cognitive factors (e.g., the spatial and temporal co-occurrence and the semantic congruence of the stimuli) contribute to the multisensory integration of auditory and visual stimuli (Vatakis and Spence, 2006, 2007; Spence, 2007).

Another corpus of research has focused on the dominance of the audio or visual modality related to music performance recordings. A study evaluating performance quality judgments of music recordings presented with only video, only audio and with both audio and video of the performances reported the dominance of visual information over sound (Tsay, 2013). The music performances used in the study were short 6s clips of the top three finalists of several prestigious music competitions. A later study expanded on this, demonstrating that when differences in performance quality were evident, participants' judgements were most accurate when evaluating the performances with only the audio, suggesting that the sight-over-sound effects in the judgments of music performance do not always hold and auditory information can inform audience response in case of clear differences in performance quality (Mehr et al., 2018).

These aspects were further analyzed in a recent study conducted by Jakubowski et al. (2020) investigating the multimodal perception of interpersonal synchronization in musical duo improvisations. Researchers in the study observed that stimuli with only video were judged less synchronized than stimuli with only audio or audio plus video of the performance, based on continuous perceptual ratings. These effects were found mostly for pulsed jazz duo improvisations. They also found relative dominance of the visual modality in predicting perceived synchrony for the stimuli displaying both audio and video. However, this depended on the stimulus duration and the ratings' type. Visual information tended to provide more cues than audio features, in line with Tsay (2013), when participants rated continuously pulsed music clips lasting on average 54.5s and non-pulsed clips that were on average 41.9s long. However, the analysis of synchronization judgments based on global ratings of short video clips (of about 10s long) implies that auditory features might be better predictors of synchronization judgements than visual aspects of the performance. Overall, these results suggest that the modality of stimulus presentation impacts judgments of music performances, depending on the quality and duration of the music performance. The evaluation of short performances might rely more on auditory information. In contrast, visual cues might contribute more to the judgment of longer recordings. Sound synchronization, in fact, manifests at lower time-scales than body motion coordination (Bishop et al., 2019b). Further research is needed to show how different modalities shape audiences' understanding of how together an ensemble is. The current study addresses this by analyzing differences in perception of togetherness between musicians in relation to the modality of stimulus presentation, comprising audio only, video only, and video and audio of the music performance.

Since visual information is clearly important, it is also valuable to understand more specifically where visual attention is directed when people are watching performances. Studies on audience gazing behavior suggest that audience visual attention is influenced by the dominance of the musical part (Kawase and Obata, 2016). In a study based on singing duo performances, it was found that the musicians singing the melody part attracted more visual attention than those performing the accompaniment (Kawase and Obata, 2016). They also found that audience gaze behavior is related to the gaze shift between co-performers, as audience attention followed when performers shifted their gazes toward the co-performer (Kawase and Obata, 2016). It remains unclear where participants would most look at when judging ensemble performances with a more balanced distribution of leadership than in the previous studies and when the eye gaze of the musicians is not visible. What attracts the audiences' gaze whilst evaluating the level of togetherness between musicians? This study investigates which parts of musicians' body audiences look most at, whilst rating togetherness between musicians in a piece featuring a balanced leadership distribution. A better understanding of the factors that attract visual attention when appreciating the level of togetherness in music performance contributes to a holistic understanding of human communication.

### 1.4. The role of music expertise on perception of interpersonal coordination in ensembles

Furthermore, another line of research analyzing human perception of music performance has studied the impact of the participants' music background. As described above, it has been shown that musically untrained listeners are sensitive to the degree of interpersonal synchronization in string quartet performances (Wing et al., 2014). The sensitivity to asynchrony can increase with training in asynchrony and order discrimination tasks (Mossbridge et al., 2006), but members of an audience are not able to discriminate differences in asynchronies between musicians in the order of 10ms in singing quintet performances, regardless of the music training of the participants (D'Amario et al., 2019). These results suggest that synchrony perception might depend on the participants' music background. Differences between musical expertise groups in continuous perceptions of the arousal of an electroacoustic piece were also found (Dean et al., 2014). Overall, these findings suggest the relevance of music expertise when judging music performance.

This aspect might also play an important role in the togetherness evaluation. Music training changes how we hear/see music by making us more sensitive to small-scale differences in musical parameters, for instance in the asynchronies' magnitude (Mossbridge et al., 2006) and pitch contours (Schon et al., 2004). Music training also changes how we understand music as a construct and what we find meaningful (Hansen et al., 2016). However, perception of togetherness in participants with varying degrees and types of music background remains unclear. Musicians might have a more complex understanding of what it means “to be together in music” than novices, for example, thinking of togetherness as partial alignment and complimentarity in how collaborating partners experience the music that they are creating rather than simply sound synchronization.

### 1.5. The current study

This research investigates the perception of togetherness in audio and video recordings of duo performances, from the perspective of an audience with varying musical expertise and instrumental background and in relation to different modalities of stimulus presentation.

Based on studies on the perception of music ensemble performance and expressiveness, we hypothesized that ratings of togetherness are related to the modality of stimulus presentation, and that the audiences' music background as well as sound intensity, a factor contributing to expressive intensity that could relate closely to togetherness, impact judgment of togetherness to some extent. Novices were especially expected to respond to certain expressive parameters such as sound intensity related to emotional arousal. In contrast, professional musicians were expected to respond to interpersonal synchronization, based on previous studies observing that the sensitivity to synchrony can increase with training (Mossbridge et al., 2006). Furthermore, we predicted that higher ratings of togetherness are related to higher strength in body coordination, based on findings showing listeners' sensitivity to leadership dynamics in large ensembles measured in terms of magnitude of multidirectional information flow, i.e., how much performers influence each other (D'Ausilio et al., 2012), and to the quantity of body motion, which is a measure of energy of the performances.

In addition to the above aims of this study, we were also interested in the eye-gaze behavior of the audience whilst rating togetherness, as this would allow us to contextualize any findings of the impact of audio and visual features of the music performances on togetherness perception. The current study was exploratory in terms of participants' visual attention whilst ratings togetherness. We could predict that visual attention of professional musicians whilst ratings togetherness is drawn to head motion rather than upper body motion, as head motion would be expected to convey expressivity and inter-performer dynamics most strongly (Goebl and Palmer, 2009; Keller and Appel, 2010; Glowinski et al., 2013; Bishop et al., 2019b), and is not involved in sound production. But, novices might look more at the upper body motion as they might find it more difficult to discern sound-producing movements of arms and hands than expressive movements of the head.

## 2. Methods

### 2.1. Participants

Thirty participants (age *M* = 24.7 years old, *SD* = 4 years; 19 females, 11 males) took part in the study. As shown in Table 1, they were classified based on their music training as: (i) *novices* (*n* = 10), comprising university students from the University of Vienna with little or no music background, or (ii) *semi-professional musicians* (*n* = 20), comprising advanced music students from mdw—University of Music and Performing Arts Vienna.

**Table 1:** Classification of participants based on their music training and instrumental expertise.

**Classification**	**Group (sample size)**		
Music background	Novices (10)	Semi-professional musicians (20)	
Instrumental expertise	N/A	Pianists (10)	Clarinetists (10)

The semi-professional musicians reported having on average 14.5 years of formal training (*SD* = 3.2 years) and practicing on average 3.5 h per day (*SD* = 0.6 h). The semi-professional group was split in 2 subgroups: pianists (*n* = 10) and clarinetists (*n* = 10) based on their instrumental experience. All participants self-reported normal hearing and three self-reported perfect pitch. They received a nominal award of 20 Euros.

The Ethics Committee at mdw—University of Music and Performing Arts Vienna approved the procedures of this study (reference EK Nr: 05/2020).

### 2.2. Stimuli

#### 2.2.1. Stimulus selection

**Figure 1:**
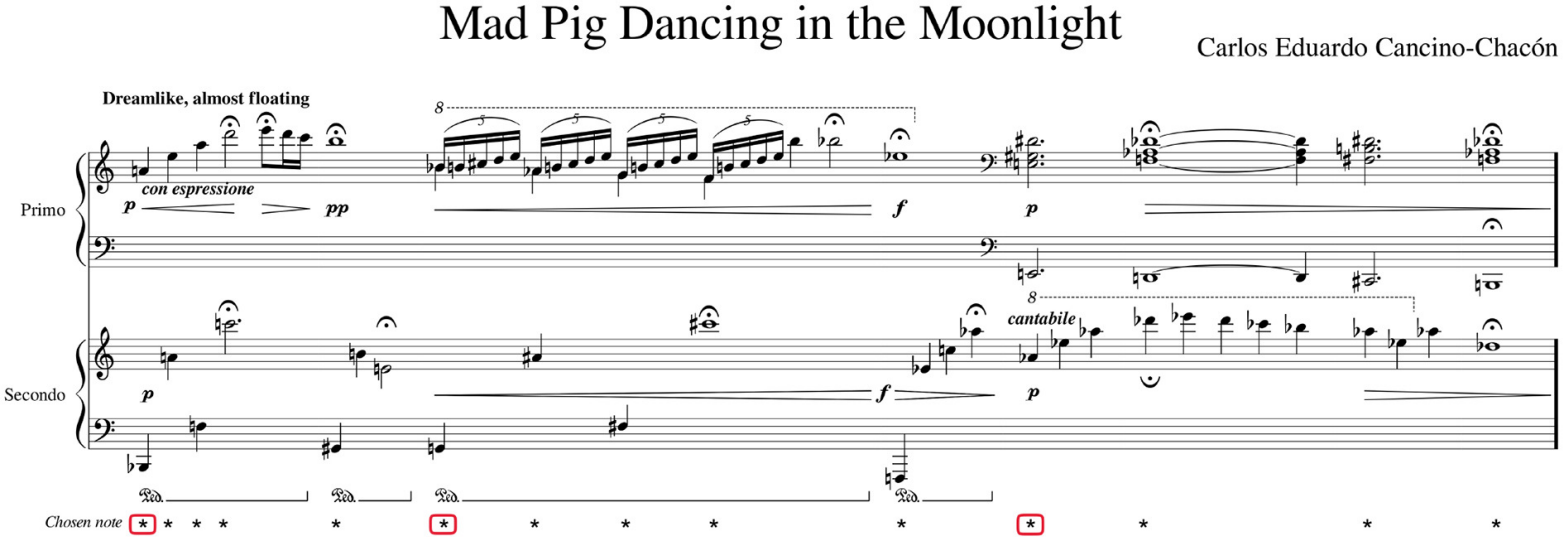
Piano duo excerpt from Bishop et al. (2019a) that was used in the current investigation. The figure displays the notes and chords, highlighted with *, upon which the analysis of interpersonal synchronization was based, as well as the initial note, highlighted with red rectangle, of the three musical phrases comprising this section. The figure is ©Bishop et al. (2019a), licensed CC-BY.

Two sets of recordings were presented to the participants for the togetherness rating task. These comprised a set with 8 piano duo recordings and a set with 8 clarinet duo recordings. These recordings were previously collected for Bishop et al. (2019b,a), which evaluated the effects of rehearsal on body coordination in musical duos. The duos in the studies recorded the same piece, composed by the second author of Bishop et al. (2019b), several times over. The current study focused on a section of the piece, shown in Figure 1, that presented a particular challenge to performers: this section has no notated meter and could be performed with free timing. This section was selected for the current study as it was expected to encourage variable experiences of togetherness. As shown in Figure 1, it comprises three main phrases: in the first two phrases, the Primo plays the melody and therefore is likely to assume the role of leader; in the third phrase, the Secondo plays the melody and is therefore likely to assume the role of leader. The first two phrases were on average 13 and 15s long, respectively, whilst the last one was longer and lasted on average 18s. The recordings of the full excerpt (i.e., all the three phrases together) were on average 47s long. Recordings of the full excerpt were presented to the participants in this study.

**Figure 2:**
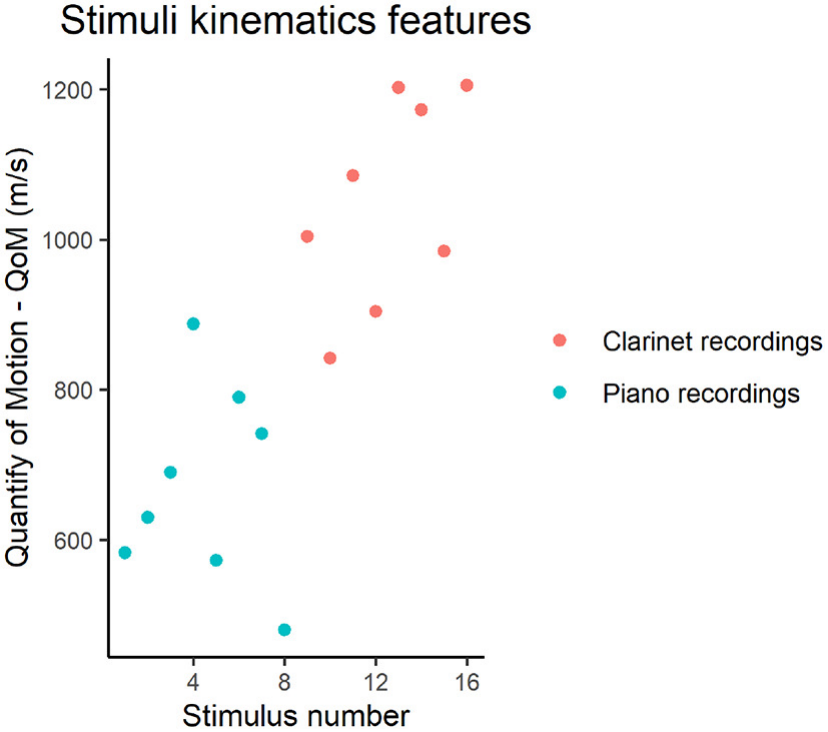
Kinematics features of the piano and clarinet recordings selected for the current study, showing the overall quantity of motion (QoM) in the y-axis for each stimulus. The QoM for the piano recordings was computed across all the markers applied on the musicians bodies; in addition to these markers, QoM for the clarinet recordings also included motion of the four markers applied on the clarinets.

The recordings in the current study were selected from the full data-set collected for Bishop et al. (2019b,a) on the basis of the overall quantity of body motion (QoM), in order to represent its full distribution. QoM was computed for all markers mounted on the musicians' bodies and for markers mounted on the clarinets. In order to compute QoM, first, raw position data recorded at 240 Hz were smoothed using the Savitzky-Golay filter^1^ with a window size of 25 frames, and the first derivative of smoothed marker positions was calculated for each marker. Second, summed velocities were computed from the Euclidean norm of 3D positions for each marker and musician per second, and then summed for each duo per second, pooling together the velocities of each marker and musician. Finally, an overall grand mean of summed velocities for each duo was computed per recording. As shown in Figure 2, this process provided a list of 16 QoM values (i.e., one per stimulus), representing the full distribution of QoM features of the original data set.

#### 2.2.2. Stimulus processing

Performance data included: (i) MIDI recordings from two Yamaha Clavinovas for piano duos; (ii) stereo audio recordings from a room microphone (Neumann KM A P48) and close-proximity microphones (DPA d: vote 4099) placed on the clarinets; and (iii) infra-red motion capture (MoCap) recordings of body and instrument markers. Audio and MIDI data were collected using a Focusrite Scarlett 18i8 sound card, and recorded as separate tracks in Ableton Live.^2^ Motion data were recorded at 240Hz using a 10-camera (Prime 13) OptiTrack motion capture system. A film clapboard with reflective markers was used to synchronize audio and motion data. Body motion data consisted of trajectories of 21 reflective markers per musician placed on the head and upper body, as follows: three markers on the head and back, three per hand, two per shoulder and arm, and one on the chest. Instrument motion was tracked using four markers per instrument. The four markers were placed at the corners of each keyboard for piano duos. For clarinet duos, two markers were mounted on a small stick across the bottom and the top of the clarinet.

The stereo recordings of the selected performances were imported into Audacity^3^, and the section of interest for this study was exported as mono wave file with 32 bit PCM at a 44.800 sampling rate. Since the level of the piano audio recordings was very low, audio rendered from the MIDI recordings was added to improve the audio experience. Audible breaths at the beginning of the performances were removed. The overall level of the audio was manually equalized across recordings so that all recordings were heard approximately at the same level, but the relative expressive dynamics of each performance remained unaltered.

**Figure 3:**
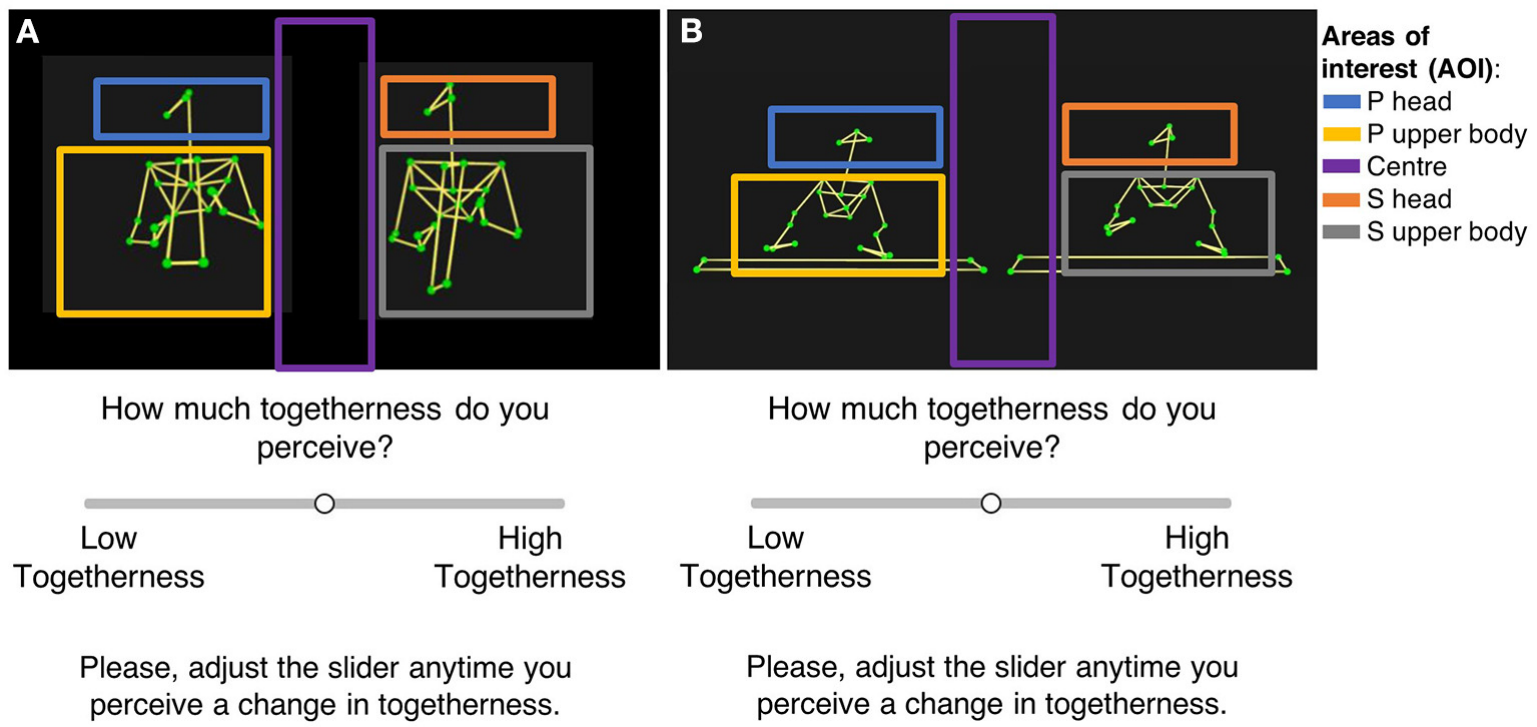
Example of the perceptual task during which participants rated how much togetherness they perceived in the clarinet performances **(A)** and piano duo performances **(B)** in a sliding scale from low to high togetherness. In both displays, the Primo (P) was on the left and the Secondo (S) on the right side of the screen. The figure also displays the five areas of interest (AOI) defined for each stimulus to analyze participants' eye-gaze during the rating task; these comprises Primo and Second head, upper body (including musicians' arms, hands, and the instrument), and the center of the screen. The AOI markers have been added to this figure for demonstration purposes and did not appear in the videos that participants saw.

MoCap data of the clarinet and piano recordings were imported into Qualysis Track Manager^4^, where visual segments (bones) were added between markers. The section of interest was exported as an .avi file at 30 frames per second (fps). The exported MoCap recordings and the audio were then imported into the video software OpenShot^5^ to create audio-only (AO), video-only (VO), and audio plus video (AV) versions of each stimulus. A visual 5-s countdown was added to all stimuli to signal the beginning to the participants. Stimuli were exported as video files (.mp4) at 720p and 30fps. These files showed the musicians from the front standing next to each other, with the Primo to the left side of the screen, and the Secondo to the right side, as shown in Figure 3.

### 2.3. Apparatus

The stimuli were presented to participants using a Desktop PC computer with an Intel Core i7-6700 3.40GHz central processing unit with 16 GB of RAM, running Windows 7. Participants wore headphones, and volume was set to a level of 75db. The headphone level was measured using an audio sine wave and a professional sound level meter placed 3 cm from the left loudspeaker driver of the headphones and pointing at its center.

The stimuli were presented to the participants through the Gorilla online platform^6^ running in Google Chrome 87.0.4280.88). Gorilla was found to achieve precision of about 10ms in reaction times (Bridges et al., 2020) on a number of browser configurations, including Google Chrome. Gorilla was set to collect slider values every 100ms (the smallest setting available), and the handle of the slider at the beginning of the recording was placed at the center of the slider.

Eye-gaze data from participants whilst completing the rating tasks were collected using SMI ETG 2 wireless glasses sampling at 120 Hz. Magnetic snap-on corrective lenses were applied over SMI glasses for the required distance correction for those participants wearing prescription glasses within the range of –4.0 and +4.0. Participants were seated approximately 80 cm from the screen, and lights level were maintained unaltered during the task.

### 2.4. Design

This study used a 2 (stimulus group: piano duo vs. clarinet duo recordings) × 8 (stimuli, i.e., performances within stimulus group) × 3 (modality of stimulus presentation: audio only, AO; video only, VO; and, audio plus video, AV) × 2 (music background: novices vs. semi-professionals) design. Stimulus group, duo performance and modality were the within-subject variables, whilst music background was the between-subjects variable. In addition, the instrumental expertise of the semi-professional musicians, being either pianists or clarinets, was a between-subject variable. The order of stimulus group was counterbalanced within music background, and the order of stimuli randomized within each stimulus group. Each recording of the selected excerpt was presented one time in each condition to each participant, except for two randomly selected clarinet and two piano recordings that were presented twice to participants for future analyses of individual consistency. Thus, each participant was presented with a total of 52 recordings (48 stimuli presented once + four repetitions of selected stimuli); the responses related to the four recordings presented the second time were not included here in the analysis of togetherness evaluation.

### 2.5. Procedure

Participants were invited to take part in a single session that took place at the Department of Music Acoustics of mdw—University of Music and Performing Arts Vienna. First, participants received spoken and written explanations of the research project and the tasks, then they gave written consent to take part in the study and filled in a background questionnaire regarding their music experience. Next, participants were presented with a description of togetherness as follows: “Togetherness is a mental state characterized by the feeling of being in full cognitive synchrony with the co-performer(s). It is often described as being in the zone, or entering a zone of magic, where things naturally flow and click, and everything becomes concerted and blended. During these optimal performance periods, a sense of individual control disappears, and musicians strike a groove together and tend to do everything together.”

Then, participants were fitted with wireless glasses tracking their eye-gaze, which were calibrated using a white board with calibration markers. Ultimately, whilst wearing their glasses, participants were asked to watch and/or listen to ensemble recordings presented on a computer screen and simultaneously rate their perception of togetherness between musicians, by moving a horizontal slider from low to high togetherness with a computer mouse, as shown in Figure 3.

Participants completed three practice trials before beginning the first stimulus group, consisting of three piano and clarinet recordings randomly selected from the pool of stimuli prepared for the study. Participants were invited to take a 2-min break between stimulus groups in order to reduce fatigue.

### 2.6. Analysis

To investigate how visual and audio musical features contribute to audiences' judgments of togetherness between musicians, an analysis procedure was carried out comprising the following three consecutive steps:

1. Extraction of visual and audio features of the duo performances (explanatory variables) as well as participants' togetherness responses (response variable), as listed in Table 2.
2. Analysis of the perception lag, since participants' slider movements were expected to lag to some extent behind the musical events.
3. Implementation of step-wise mixed modeling to investigate the impacts of visual and auditory features on the judgment of togetherness, and testing also the effects of modality of stimulus presentation and participants' background, as shown in Table 3).

**Table 2:** List of the explanatory and response variables of the study, including the related cues to togetherness, the metrics computed, and the data-set from which they have been extracted.

**Variable**	**Cue**	**Metric**	**Data-set**
Explanatory	Visual	QoM	Motion capture recordings
Explanatory	Visual	CWT power	Motion capture recordings
Explanatory	Auditory	Sound intensity	MIDI recordings
Explanatory	Auditory	Note onset synchronization	Audio and MIDI recordings
Response	Perception	Togetherness ratings	Slider response
Response	Eye-gaze	Fixation	Eye-tracking data

Onset synchronization of the piano performances was estimated using the MIDI recordings, whilst that of the clarinet performances was computed using the audio recordings.

**Table 3:** Generalized linear mixed models fitted in the study to analyse the impact of sound intensity and body motion on togetherness perception by modality of stimulus presentation (i.e., AO, audio only; VO, video only; AV, audio and video) and participants classification (i.e., a, semi-professional musicians; b, novices; c, pianists; d, clarinetists).

**Model *n***	**Fixed effect(s)**	**Random effects**	**Data-set**
1	Sound intensity		
a b c d		Participants Participants Participants Participants	AO, semi-professional musicians AO, novices AO, pianists AO, clarinetists
2	QoM, CWT measures		
a b c d		Participants Participants Participants Participants	VO, semi-professional musicians VO, novices VO, pianists VO, clarinetists
3	QoM, CWT measures, sound intensity		
a b c d		Participants Participants Participants Participants	AV, semi-professional musicians AV, novices AV, pianists AV, clarinetists

Details of the three steps are reported in Sections 2.6.1–2.6.5, respectively. In addition, details of the analysis of the eye-gaze behavior of participants whilst completing the rating task are also provided at the end (see Section 2.6.6).

#### 2.6.1. Visual cues: Quantity of motion and between-players coordination

The analysis tested the impact of: (i) coordination in body acceleration and (ii) quantity of body motion (QoM) on togetherness ratings. Strength of coordination was operationalized in terms of the power of common periodic oscillations in musicians' motion, calculated using cross wavelet transform analysis. Coordination of body motion was analyzed for a total of six paired markers placed on the chest, front head, left and right shoulder and arm of each musician (e.g., chest of the Primo with that of the Secondo, etc...). Hand markers were excluded from the coordination analysis as hand motion is more tightly linked to sound production and largely dictated by the score.

In order to measure the strength of coordination, first, acceleration for each marker was computed as the second derivative of smoothed marker positions. They were then subject to cross-wavelet transformation (CWT), using the R package “WaveletComp” (Roesch and Schmidbauer, 2018) with the complex-valued Morlet wavelet as mother wavelet. The range of periods to be considered was decided based on the structure of the music, which comprised three phrases, each lasting about 15s. The selected periods ranged from 0.3 to 7s, corresponding to the mean duration of the semi-phrase. Time-series data for the period within this range that had the highest power was extracted for each stimulus and down-sampled to 10Hz, in order to match the sampling rate of the togetherness response data.

For QoM, two related measures were calculated: (i) total QoM calculated for each duo across all markers and (ii) local QoM computed for the subsets of markers that were selected for the CWT power analysis, i.e., head, right and left arm and shoulder, and chest. Total QoM was computed by summing QoM values across markers at each timestamp, for each musician, then averaging per timestamp across duo partners, to obtain one average series of summed QoM values per duo. Total QoM for the clarinet recordings also included motion of the markers on the clarinets. Local QoM was calculated by averaging QoM per timestamp, across duo partners, for each of the selected markers (i.e., head, chest, and right and left arm and shoulder). Both total and local QoM were sampled at 10Hz, in line with the togetherness ratings.

It was of interest to test how QoM and strength of coordination differed between body parts (i.e., head, chest, left/right arm/shoulder). This was achieved using step-wise linear mixed models. Body part was entered in the model as the explanatory variable, and time-series CWT power data and local QoM data were input as response variables. Stimulus number was fitted in the model as a random effect; times nested in stimuli were also entered in the model as auto-regressive errors to address temporal autocorrelation and potential endogeneity within each stimulus across time.

#### 2.6.2. Auditory cues: Sound intensity and onset asynchronies

The effects of auditory cues on togetherness ratings were investigated for two auditory parameters: (i) sound intensity and (ii) interpersonal synchronization. Sound intensity was calculated as the root-mean-square (RMS) of the audio recordings. Values were extracted at 10Hz with a rectangular window and 50% overlap, since the signals were periodic and all points were equally weighted. Sound intensity data were extracted in Python using Madmom (Böck et al., 2016).

Interpersonal synchronization was calculated for onsets of notes and chords that, according to the music score, were supposed to be performed simultaneously. Notes of interest for the synchronization analysis (including the chords) are indicated in Figure 1. For the piano recordings, note onset timestamps of the chosen notes/chords were extracted from the MIDI recordings, and note asynchronies were computed by subtracting the onset time of the Primo from that of the Secondo. Therefore, positive asynchronies indicate that the Primo was leading, and negative asynchronies indicate that the Primo was lagging. In case of the piano chords (i.e., notes 11, 12, 13, 14 highlighted in Figure 1), average asynchronies were computed by referring to the latest onset (within each chord) of each musician (i.e., Primo and Secondo) and then by subtracting the timestamp of the last onset of the Primo from the timestamp of the last onset of the Secondo.

For clarinet recordings, onset times were first manually labeled by one of the authors (LB, with extensive experience on onset estimation) using Praat based on the waveform and spectrogram of the audio recordings, with a 2s window display. Then, perceptual onset timestamps were estimated at 70% of the max RMS with 10ms windows. Asynchronies were eventually computed as for the piano recordings, by subtracting the onset timestamp of the Primo from that of the Secondo.

#### 2.6.3. Ratings of togetherness

The togetherness ratings (the response variable) and corresponding timestamps were recorded *via* the Gorilla platform as dense data: values were reported each time participants moved the sliders, and no values were recorded when the slider remained stationary. For this reason, the exported response data were de-sparsed. A constant interpolation was used to obtain slider values evenly sampled at 10Hz, since Gorilla was set to collect slider values every 100ms, which was the smallest setting available. This step produced a list of togetherness ratings per stimulus/duo sampled at 10Hz.

Two participants reported no change in togetherness (i.e., did not move the slider) in 10 performances. Participants reported on average 46.6 changes per stimulus (*SD* = 40.4), and on average 1 change every 1.11 s. An analysis of the logs from the Gorilla online platform showed that eight of the 1568 trials collected in total during the experiment [i.e., 30 (participants) × 52 (valid trials) + 8 (partial trials)] were loaded twice, because of loading issues during the first attempt. These eight partial trials were excluded from the analysis. An investigation of the total duration of the responses for each stimulus was also computed as the difference between the true duration of each stimulus and the duration of the responses to each stimulus that Gorilla recorded. This difference was on average 144.9 ms (*SD* = 30.6*ms*), and might have been induced by initial delays in the visual display due to the Internet connection or the computer processor. This discrepancy is considered negligible in light of the total duration of the entire stimulus.

#### 2.6.4. Rating lag response

Togetherness ratings were expected to lag about 1 to 3s behind changes in auditory and visual signals, in line with literature investigating continuous response to musical events (Schubert and Dunsmuir, 1999; Schubert, 2004; Dean and Bailes, 2010). To estimate a more exact lag time, three mixed linear models per modality condition (i.e., AO, VO, and AV) were implemented. Togetherness ratings lagged by 1s, 2s and 3s comprised the response variables. The explanatory variables were: sound intensity for the AO condition; QoM and CWT power data for the VO condition; and sound intensity, QoM, and CWT power data for the AV condition. Since sound intensity, QoM and CWT power data were highly positively skewed, they were log transformed for the models.

Multicollinearity of model terms related to QoM and CWT per paired markers for the VO and AV condition was tested using the performance (Ludecke et al., 2021) package in R. The variance inflation factor (VIF), which measures the correlation and strength of correlation between the predictor variables in a regression model, was computed for each model. The results demonstrate that VIF was very low for the CWT power measures, but moderate and high for QoM computed per paired markers. For this reason, in each model, total QoM (i.e., summed across all chosen body markers) rather than local QoM (i.e., computed per paired marker) was entered as a fixed effect along with the CWT power measures.

In each model, times were entered within stimuli as first-order auto-regressive errors, and participants were included as random effects. Stimuli number was not entered in the models because the variance was negligible.

Then, to evaluate the three different models related to the three different lags, the K-fold cross-validation (CV) method, widely adopted as a model selection method (Jung, 2017), was implemented with *K* = 10, (*K*- 1) folds of the data used for model construction and the hold-out fold allocated for model validation. In light of the nested nature of the data (i.e., participants fully crossed within all variables), folding was done by participants. Model accuracy was then estimated by computing the average Mean Absolute Error (MAE) and Root Mean Square Error (RMSE) across folds. The model with the lowest MAE and RMSE was selected as the best model.

#### 2.6.5. Impact of expertise, stimuli, and auditory and visual cues on togetherness ratings

After the analysis of the visual, auditory, and perceptual features of the ensemble performances, and the estimation of the most accurate perception lag, the analysis moved to the investigation of changes in togetherness ratings. First, the simple effects of music background, modality, and stimulus group as well as their interactions on togetherness ratings were investigated through a two-way ANOVA on the whole data set. Then, an additional two-way ANOVA was conducted to analyse the simple effects of instrumental expertise, stimulus group, and modality on togetherness ratings. In both ANOVAs, togetherness ratings were entered as mean values for each recording rated.

Three model groups were implemented to test the fixed effects of (i) sound intensity in the AO recordings (see Table 3, Models 1a-1d); (ii) body motion (Models 2a-2d) in the VO recordings; and, (iii) sound intensity and body motion in the AV recordings (Models 3a-3d). Within each model group, model (a) included data from semi-professional musicians, model (b) included data from novices, and models (c) and (d) included data from pianists and clarinetists, respectively.

Explanatory variables (i.e., sound intensity, QoM, and CWT measures) were entered in the models as log transformed data. All of the explanatory variables (i.e., togetherness ratings, QoM, CWT power, and sound intensity) were entered as continuous variables. Togetherness ratings were entered in the models as lagged data, with lag time corresponding to the most accurate model resulting from the k-fold validation (see analysis description above). Participant number was entered as random intercept; stimulus number was not entered as a random effect in any model as the variance was negligible. In addition, the random effects of time nested within stimuli with first-order auto-regressive errors were fitted in the models to address temporal autocorrelation and potential endogeneity within each stimulus across time. Following Bonferroni correction, the alpha level for each model was set at α = 0.0125 (four mixed models per modality).

In addition to the models above, the impact of interpersonal synchronization (explanatory variable) on togetherness perception (response variable) was investigated. This was done for individual modality conditions (i.e., AO and AV) using data for semi-professionals and novices in separate models, and for pianists and clarinetists in separate models. Participant number was entered in each model as random intercept, whilst the effects of the chosen note number was negligible and for this reason was not entered in the model. Importantly, whilst sound intensity, QoM and CWT measures were entered in the previous models as continuous variables, onset asynchronies are discrete values. In order to analyse the impact of synchronization on togetherness response, these two measures had to be temporally aligned; for this purpose, the last note onset time of each chord was used as a reference point, and togetherness ratings were taken that lagged behind each reference point by the duration dictated by the most accurate perceptual model resulting from the k-fold validation analysis presented in the previous section.

Generalized linear mixed modeling was implemented using the glmmTMB package (Brooks et al., 2017) in R. Assumptions of linearity and homoscedasticity were checked for the residuals using the DHARMa package in R; results show that assumptions were met.

#### 2.6.6. Fixation times

Having analyzed changes on togetherness perception for some visual and auditory features of the recordings, it was of interest to analyse the eye-gaze behavior of the participants who completed the rating tasks. We carried out post-processing in SMI BeGaze. Five areas of interest (AOI) were manually defined for each stimulus video: (1) Head of the Primo, (2) Upper body and hands of the Primo, (3) Head of the Secondo, (4) Upper body and hands of the Secondo, and (5) Center (see Figure 3). Beginnings and endings of each stimulus were manually annotated in the eye-tracking takes. Percentage of total fixation time for each AOI was computed and extracted with 1 s bins. Average fixation times were then computed for each stimulus/participant/AOI. Finally, mean fixation values (response variable) were fit in a linear mixed model to estimate the fixed effects of music training and AOI, with random effects of stimuli and participants.

## 3. Results

This section reports first the results of the music performance features (see Section 3.1), analyzed to contextualize the findings regarding changes of perception of togetherness based on performance cues, then presents the results of the impact of the external music performances' features (i.e., participants' background and expertise, modality of stimulus presentation and stimulus group, see Section 3.2) and the performance cues (i.e., visual and auditory cues, see Section 3.3) on togetherness ratings. This section concludes with the findings related to the eye-gaze analysis (see Section 3.4).

### 3.1. Music performances features

**Figure 4:**
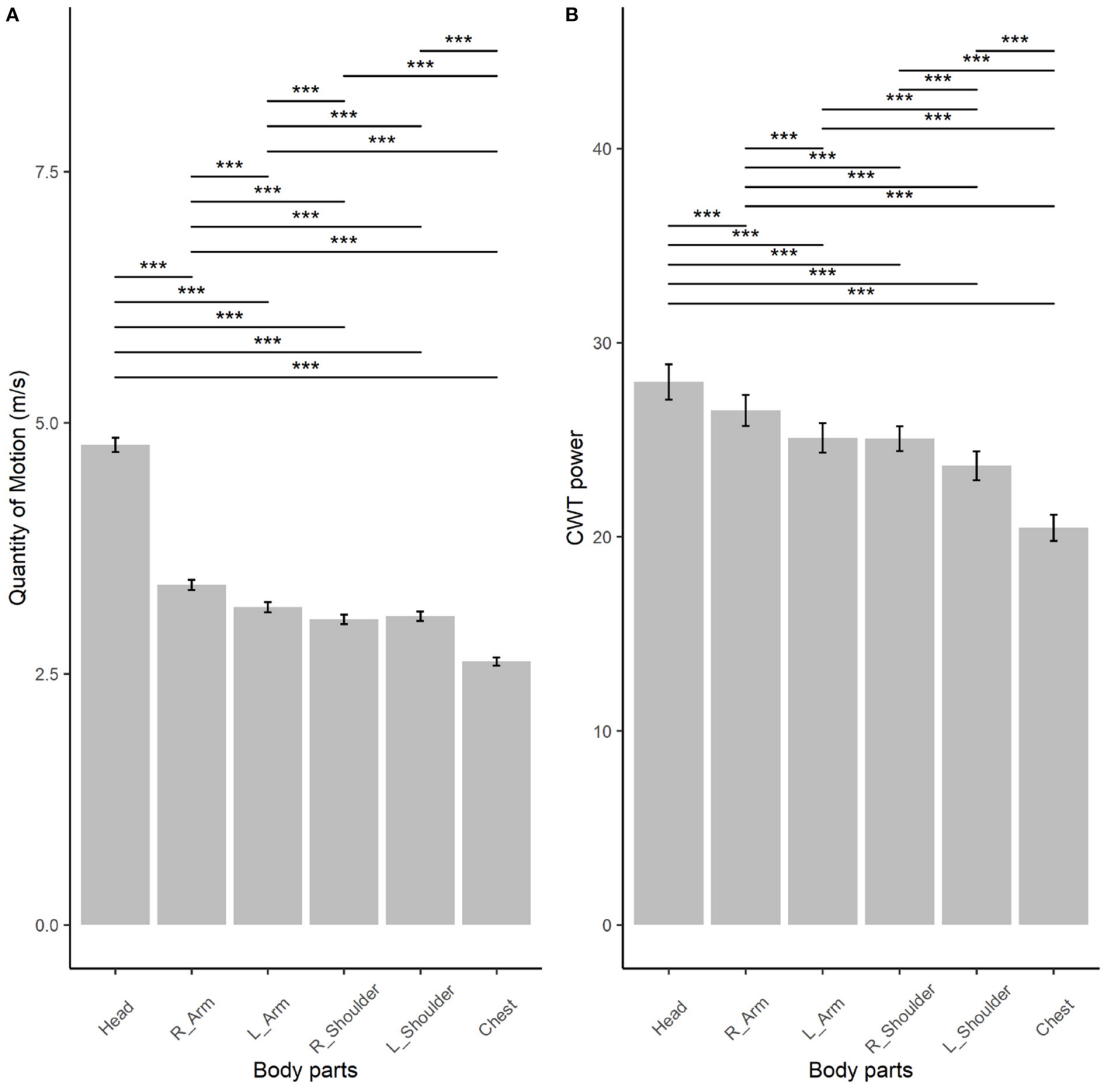
Quantity of motion (QoM, **A**) and cross-wavelet transforms (CWT, **B**) power by body parts (i.e., head, right and left arm [R_Arm and L_Arm] and shoulder [R_Shoulder and L_Shoulder], and chest), based on linear mixed modeling. Error bars represent 95% CI of the mean. *P*-values were adjusted using the Tukey *post-hoc* tests. ^***^p < 0.001.

As shown in Figure 4, both QoM (Figure 4A) and CWT power (Figure 4B) differed significantly across body parts. Interestingly, they were both highest in head motion, followed by the right arm motion. Chest motion was lowest in QoM and CWT power.

For interpersonal synchronization, mean absolute asynchronies were 127 ms (*SD* = 221 *ms*) in the piano recordings and 137 ms (*SD* = 207 *ms*) in the clarinet recordings. These asynchronies are larger than those that arise in music with less temporal flexibility and a steadier beat (Keller, 2014).

### 3.2. Impact of external music performances' features on togetherness ratings

**Figure 5:**
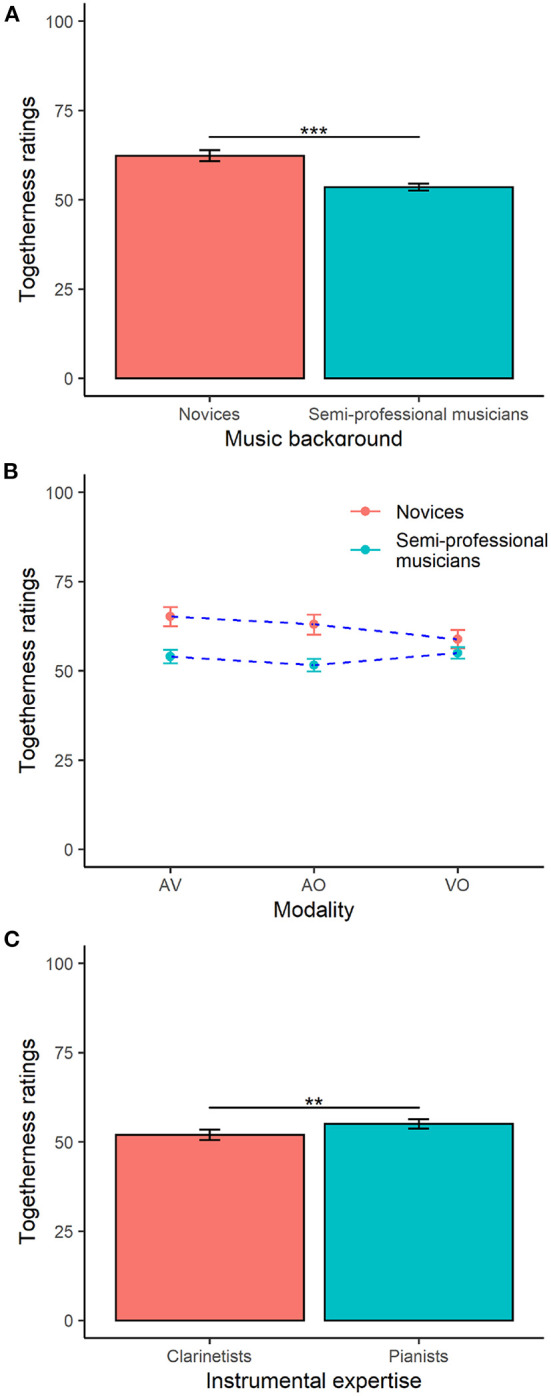
Ratings of togetherness by music background [**(A)**, novices vs. semi-professional musicians]; the interactions **(B)** between modality of stimulus presentation (audio plus video [AV], video only [VO] and audio only [AO]) and music background; and, instrumental expertise of the semi-professional musicians [**(C)**, clarinetists vs. pianists]. Maximum and minimum values on the y-axis have been fixed to allow comparison between the graphs against the full range of the scale. Error bars represent 95% CI of the mean. ^**^p < 0.01; ^***^p < 0.001.

Simple main effects analysis from the two-way ANOVA showed that music background had a significant effect on togetherness ratings [*F*_(1, 1428)_=95.3, *p* < 0.001], whilst modality of stimulus presentation and stimulus group were non-significant. As shown in Figure 5A, novices rated the stimuli as more together than did the semi-professional musicians. The ANOVA also revealed a significant interaction between music background and modality of stimulus presentation [*F*_(2, 1428)_=7.5, *p* < 0.001], but there were no significant interactions between any remaining pairs of effects (i.e., stimulus group and modality, music background and stimulus group, or stimulus group, music background and modality).

Tukey's HSD test for multiple comparisons found that novices' ratings of AV and AO recordings were significantly higher that those of the semi-professionals related to the same modality [*p* < 0.001, 95*%CI*=[−15.6, −6.7] and *p* < 0.001, 95*%CI*=[−15.8, −6.9], respectively]. Interestingly, novices' ratings of VO stimuli was not different than semi-professionals' ratings of AV and VO stimuli (see Figure 5B), suggesting that the differences in ratings between novices and semi-professional musicians relied on presence/absence of the audio modality.

An additional two-way ANOVA on the subset of data comprising only the semi-professional musicians showed that the instrumental expertise of the musicians and the modality of stimulus presentation had a significant effect on togetherness ratings [*F*_(1, 948)_=9.4, *p* < 0.01 and *F*_(2, 948)_=4.2, *p* < 0.05, respectively]. As shown in Figure 5C, pianists rated the recordings as more together than did clarinetists. There was no overall effect of stimulus group, and no significant interactions between modality of stimulus presentation, instrumental expertise and stimulus group.

### 3.3. Impact of performance cues on togetherness ratings

#### 3.3.1. Lag response

**Figure 6:**
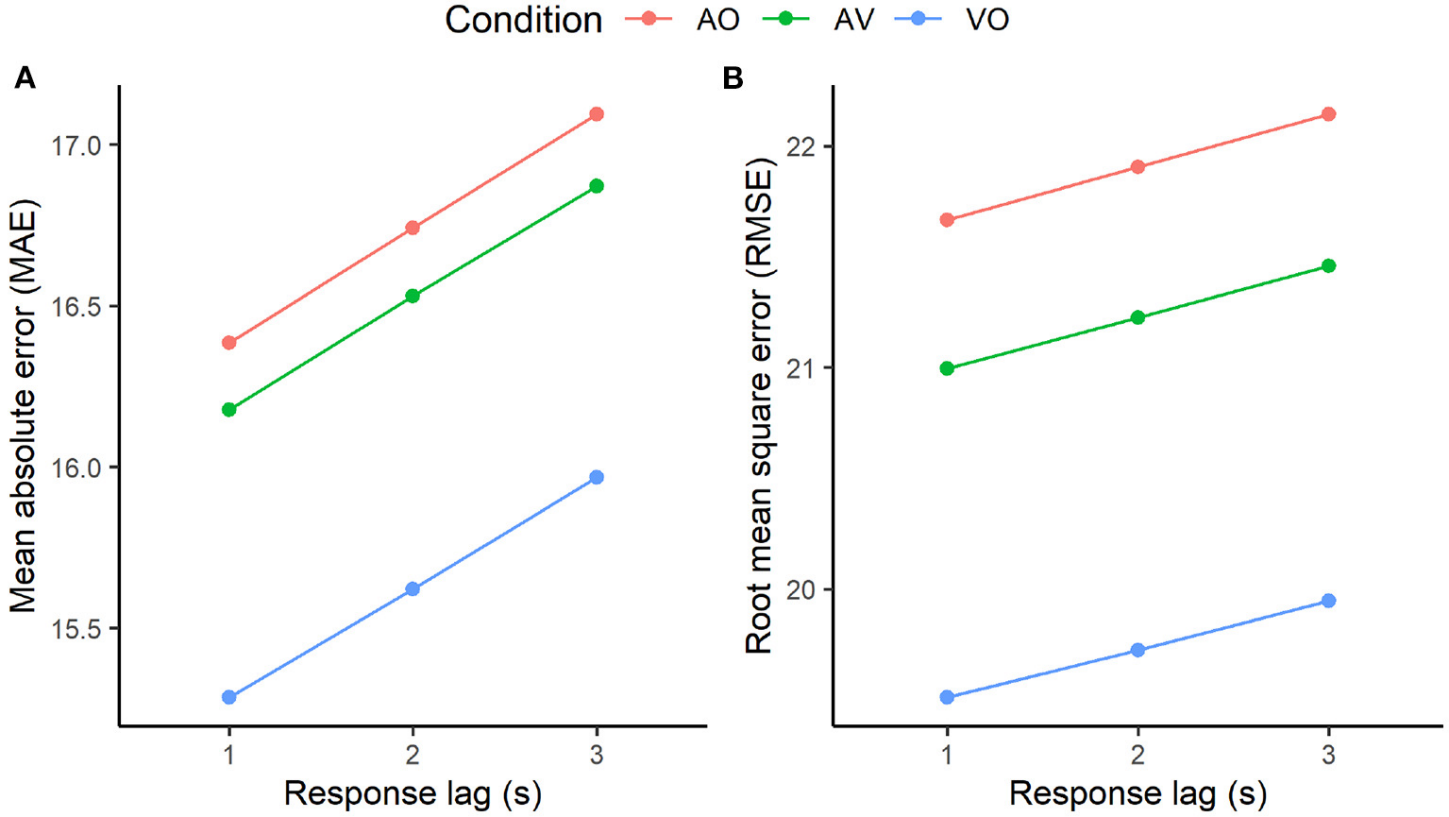
K-fold cross-validation accuracy of models computed at different response lag times (i.e., 1, 2, and 3 s lag) for the audio only (AO), video only (VO), and audio plus video (AV) conditions. Model accuracy was measured in terms of mean absolute error (MAE, **A**) and room mean square error (RMSE, **B**).

The k-fold cross validation conducted for each condition in order to estimate model accuracy demonstrated that the three models, implemented to evaluate the response time of the togetherness ratings, performed better at lag 1, although with a decimal point of difference, as shown in the accuracy measures in Figure 6. Based on these results, the analysis of the impact of visual and auditory cues on togetherness ratings was conducted accounting for a 1s lag of the togetherness ratings.

#### 3.3.2. Sound intensity and body motion

Togetherness ratings of pianists, clarinetists and semi-professional musicians were not predicted by the sound intensity of the AO and AV stimuli (see models 1a,c,d and 3a,c,d in Tables 3, 4). Interestingly, novices' ratings of AO and AV stimuli related positively to sound intensity: the higher the sound intensity, the more together the recordings were judged [model 1b in Table 4: β = 3.8, 95*%CI*[1.5, 6.1], *t*_(72420)_ = 3.2, *p* < 0.01; and, model 3b in Table 4: β = 3.7, 95*%CI*[1.3, 6], *t*_(74020)_ = 3.1, *p* < 0.01, respectively].

**Table 4:** Overview of the generalized mixed models displaying *β* coefficients and significance measuring the relationship between performance cues (i.e., auditory and visual cues) and togetherness ratings for each modality (i.e., AO, audio only; VO, video only; and AV, audio+video) and each participant group (i.e., semi-professional musicians, novices, pianists, clarinetists, and all participants).

**Condition**	**Predictors**	**Semi-pro**	**Novices**	**Pianists**	**Clarinetists**	**Model *n***
**AO**						
	Sound intensity	ns	3.8^**^	ns	ns	1a-d
	Synchronization	ns	ns	ns	ns	4a-d
**VO**						2a-d
	QoM	ns	ns	ns	ns
	Head	ns	ns	ns	ns
	Chest	ns	ns	ns	ns
	L Shoulder	ns	ns	ns	ns
	R Shoulder	ns	ns	ns	ns
	L Arm	ns	ns	ns	ns
	R Arm	ns	0.7^**^	ns	ns
**AV**						
	Sound intensity	ns	3.7^**^	ns	ns	3a-d
	Synchronization	−0.005^***^	ns	ns	−0.006^**^	5a-d
	QoM	ns	ns	ns	ns	3a-d
	Head	ns	ns	ns	ns	3a-d
	Chest	0.5^**^	ns	0.5^**^	ns	3a-d
	L Shoulder	ns	ns	ns	ns	3a-d
	R Shoulder	ns	ns	ns	ns	3a-d
	L Arm	ns	ns	ns	ns	3a-d
	R Arm	ns	0.6^**^	ns	ns	3a-d

The table also displays the model number used for the analysis of the continuous variables (i.e., 1 and 4 for AO stimuli, 2 for VO stimuli, and 3 and 5 for AV stimuli; “a” for models regarding semi-professional musicians, “b” for the novices, “c” for the pianists, and “d” for the clarinetists). The auditory cues were sound intensity and synchronization whilst visual cues were: total quantity of motion (QoM) and CWT power measures related to head, chest, left (L) and right (R) shoulder and arm. Semi-pro, semi-professional musicians; ns, non-significant;

** *p* < 0.01;

*** *p* < 0.001.

In addition, novices' ratings of VO stimuli related positively to the CWT power of the right arm: the greater the synchronicity in right arm acceleration, the more together the recordings were rated [model 2b, Table 4: β = 0.7, 95*%CI*[0.3, 1.1], *t*_(74020)_ = 3.2, *p* < 0.01); conversely, semi-professional musicians did not rate togetherness in VO stimuli based on body motion (model 2a in Tables 3, 4].

Furthermore, novices' ratings of AV stimuli also related positively to right arm synchronicity: the higher the coordination in common periodicities, the higher the togetherness ratings [model 3b, Table 4: β = 0.6, 95*%CI*[0.2, 1], *t*_(74020)_ = 2.8, *p* < 0.01]. Interestingly, pianists' ratings of AV stimuli related positively to chest motion: the higher the coordination in common periodicities in chest acceleration trajectories, the higher the togetherness ratings [model 3c, Table 4: β = 0.5, 95*%CI*[0.1, 0.9], *t*_(74020)_ = 2.5, *p* < 0.01].

#### 3.3.3. Sound synchronization

Interpersonal synchronization was a significant predictor of togetherness ratings only for semi-professional musicians, not for novices. Specifically, the smaller the asynchronies of the AV stimuli, the more together the performances were perceived by the semi-professional musicians [β = −0.005, 95*%CI*[−0.01, 0.00], *t*_(4320)_ = −3.5, *p* < 0.001]. Interestingly, these results did not rely on the pianists' judgement, but on that of the clarinetists' evaluation [β = −0.006, 95*%CI*[−0.01, 0.00], *t*_(4320)_ = −3.0, *p* < 0.01].

### 3.4. Eye-gaze

**Figure 7:**
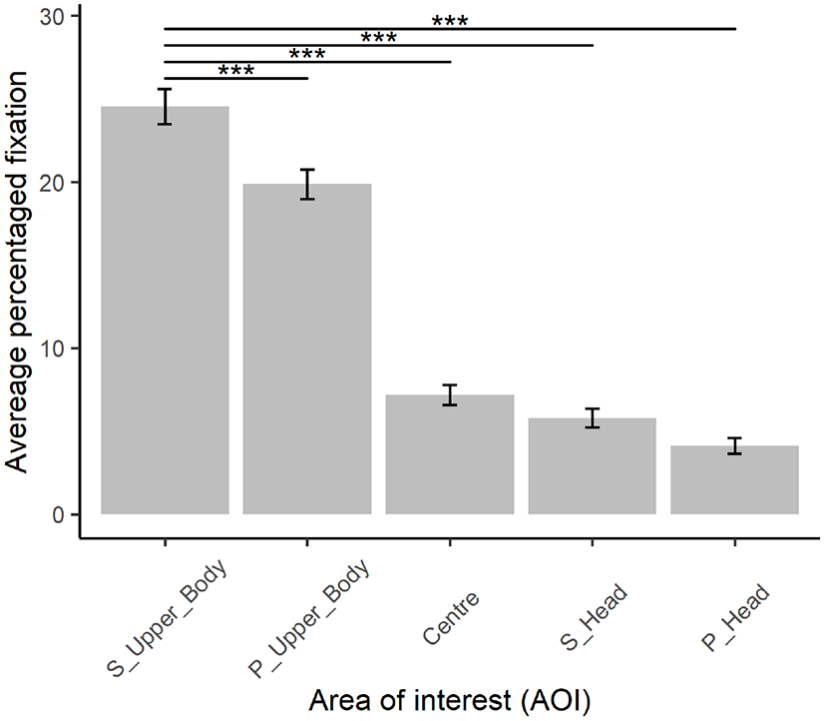
Distribution of visual attention across different areas of interest (AOI): S_Upper_Body (upper body of the Secondo), P_Upper_Body (upper body of the Primo), center of the screen, S_Head (head of the Secondo) and P_Head (head of the Primo). Error bars represent 95% CI of the mean. ^***^p < 0.001.

Participants spent most time looking at the upper body of the Secondo and their instrument; then, in descending order, participants looked at the upper body of the Primo and their instrument [β = (−4.7), *t*_(45879)_ = −8.9, *p* < 0.001], the center of the screen [β = (−17.4), *t*_(45879)_ = −32.9, *p* < 0.001], the head of the Secondo [β = (−18.8), *t*_(45879)_ = −35.5, *p* < 0.001], and the head of the Primo [β = (−20.4), *t*_(45879)_ = −38.7, *p* < 0.001] (see Figure 7). The music training of the participants did not predict how visual attention was distributed across AOIs.

## 4. Discussion and conclusion

This study investigated the real-time, continuous judgement of togetherness in musical duos by an audience with varying music background. Ratings of togetherness were analyzed in relation to certain visual and auditory features of the music performances as well as the modality of stimulus presentation. Audio only (AO), video only (VO) and audio plus video (AV) modalities were tested. In addition, the eye-gaze behavior of the participants whilst rating togetherness was also analyzed, in order to contextualize any findings related to the togetherness response.

Audience perception of togetherness was found to be heavily informed by the music background of the participants and its interaction with the modality of stimulus presentation. Novices in our study generally rated the performances more together than the semi-professional musicians. This is somewhat in contrast to results reported by Jakubowski et al. (2020), who found that synchronization ratings for non-pulsed music were slightly positively correlated with the numbers of years of musical training. Overall, musical training may play a different role in the perception of synchronization than that of togetherness. Notably, in our study, novices' ratings of togetherness in AV and AO stimuli were higher than the semi-professional musicians' ratings of AV, AO, and VO stimuli, but novices' ratings of VO did not differ than those of the semi-professional musicians. This implies the relevance of the audio information in the togetherness evaluation of novices and semiprofessional musicians. These results are somewhat also in line with previous studies on synchronization judgements observing higher ratings of synchrony in the evaluation of AO and AV stimuli than VO recordings (Jakubowski et al., 2020).

The study also showed that novices' judgment of togetherness between musicians was positively related to sound intensity, in both AO and AV recordings: the higher the sound intensity, the more together novices judged the recordings. Previous studies on listeners' perception of sound intensity demonstrate that this feature is perceived in terms of physical effort and physical expression of the performance (Olsen and Dean, 2016). Novices in this study responded to perceived loudness as they might have understood togetherness in terms of increasing emotional intensity, or loudness might have been the most noticeably changing feature for them. Conversely, semi-professional musicians did not respond to sound intensity when rating the AV recordings, but to note-to-note synchronization, a factor that often contributes to performance excellence in ensemble playing. This effect arose mostly for the clarinetist listeners. This result is also somewhat in line with Jakubowski et al. (2020) observing that mean synchrony ratings were positively related to the years of musical training of the listeners, i.e., the higher the number of years of musical training, the higher the level of synchrony between musicians was judged.

Overall, these results imply that trained musicians are sensitive to asynchrony in music, and that precision in perception of asynchrony increases with training (Mossbridge et al., 2006) and affects judgments of togetherness. Novices associate togetherness with loudness, maybe because loudness changes are relatively easy to discern. Future studies might also investigate to what extent the relationship between synchronization and togetherness ratings depend on the style of the music and the artistic intentions of the musicians. Listeners might also rate as highly together music that explicitly avoids a tight synchronization between musicians for expressive reasons, for example, to increase grooviness (Skaansar et al., 2019).

Interestingly, novices also rated the VO and AV recordings based on the coordination of the right arm motion: the greater the synchronicity in common periodicities of acceleration trajectories of the right arm, the more together recordings were rated. Quantity of body motion (QoM) and similarity in acceleration trajectories were highest for head motion, followed by right arm motion. The latter was part of the upper body area where participants spent more time looking whilst rating togetherness. Overall, these results suggest that novices were informed by similarity in right arm coordination as they were looking at body parts with higher QoM and with more similar coordination. Conversely, semi-professional musicians rated AV recordings in relation to the chest, which represents the body center: the higher the coordination in common periodicities in chest acceleration trajectories, the higher the togetherness ratings. Taken together these results imply that semi-professional musicians sought information in the overall quality of coordination of the body motion, whilst novices were informed by individual body parts that had the highest QoM and coordination power, such as right arm motion.

Whilst perception of togetherness in AO and VO stimuli was related to sound intensity and right arm motion, perception in the AV stimuli was related to sound synchrony and chest motion (for semi-professional musicians) and sound intensity and right arm motion (for novices). This suggests that perceptions of togetherness are shaped in complex ways by the different information that is available about a performance. The fact that both auditory and visual features of the performances contributed significantly to perceived togetherness in the AV recordings further expands Jakubowski et al. (2020) in which the authors demonstrated, in line with studies on performance quality (Tsay, 2013), that some aspects of the visual information (i.e., total QoM and energy of the similarity in common periodicities of body motion) were better predictors of continuous synchrony ratings than certain auditory cues (i.e., event density, pulse clarity and spectral flux). Taken together, these results suggest the relevance of auditory and visual information in the perception of interpersonal synchronization and feelings of togetherness between musicians in ensembles.

Contrary to our prediction, quantity of body motion did not contribute to the perception of togetherness, but in other research this was found to be a predictor of perceived synchrony (Jakubowski et al., 2020). The different material used for the study might explain these results. Another explanation might be that the overall quantity of motion indeed does not contribute to togetherness judgments but to perceived synchrony, and that participants judged togetherness more in terms of quality (and therefore they responded to the similarity of certain body parts' coordination) than quantity of motion.

This study also analyzed the perception of togetherness in relation to the eye-gaze behavior of an audience whilst rating togetherness. Results showed that our participants looked most at the Secondo upper body and their instrument. Participants' attention to body rather than head motion suggests that they may have sought information where there was more motion. Their attention might have been drawn instead to the players' heads if the players' faces had been visible. Future studies might replicate this investigation by having musicians' full body visible, rather than just skeletons, and further investigate whether periods of togetherness are associated with players' faces. Participants' visual attention to the Secondo might be due to the fact that the Secondo was placed on the right side of the screen, which participants might assume indicates a more important role.

In this study, we investigated how audience members judged how much the musicians experienced togetherness, and we measured this by relating audience ratings to certain objective measurements that might be informative of togetherness between musicians (i.e., sound synchronization and body motion similarity). Future investigation might replicate this study considering also musicians' perception of togetherness in parallel to the audience perspective of togetherness. A mixed design allowing the triangulation of musicians' and audiences' perceived togetherness as well as togetherness measured in body motion and sound recordings is currently underway in our lab and should shed more light on the relationships between subjective and objective measurements of togetherness from the perspective of the musicians as well as the audiences.

Some limitations of the study should be noted. First, in this study, participants were presented with reduced stimuli comprising only audio and body motion, without other potentially useful cues like facial expressions or gaze. This was done to investigate systematically whether body motion would inform audience evaluation of togetherness. Future studies might replicate this study with a more ecological recordings, showing musicians' face too.

Second, the study does not allow for conclusions to be drawn about how the different temporal resolutions of auditory and visual processing affect togetherness judgements. Timing is perceived more precisely in auditory signals than in visual signals (Hove et al., 2013). Correspondingly, the same absolute magnitude of asynchrony might be more readily noticed in musicians' sound output than in their periodic body motion. It remains an unresolved question how audiences integrate information about auditory and visual synchrony in music performances, especially if, for example, the performers look synchronized despite asynchronies in their sound being audible. The current study was not intended to investigate this sort of conflict; indeed, to do so, it would be necessary to construct very controlled stimuli where auditory and visual synchrony were independently manipulated. However, our finding that both sound and visual coordination contribute to judgments of togetherness lays the groundwork for future studies, which may investigate how these cues to togetherness interact.

To conclude, this study contributes to a better understanding of how togetherness is judged. By building on literature investigating togetherness from the perspective of the performer (Sawyer, 2006; Hart et al., 2014; Hart and Di Blasi, 2015; Gaggioli et al., 2017) and focused mainly on self-reported experiences of togetherness, this study shows that togetherness judgements are multimodal and shaped by several interacting factors, including both auditory and visual features of the performances. Sound intensity, sound synchronization, and similarity in body motion coordination play major roles in the evaluation of togetherness. This complex construct can be meaningfully understood by novices, even if in a different way than the experts. The results of this study advance our understanding of the visual and auditory cues that contribute to the perception of togetherness between musicians in ensemble playing. These findings also provide a valuable contribution to social psychology by clarifying the perceptual mechanisms involved in socio-cognitive judgments of human interactions and coordination.

## Data availability statement

The datasets, including stimuli and the togetherness ratings, can be found at https://doi.org/10.5281/zenodo.7128672.

## Ethics statement

The Ethics Committee at mdw—University of Music and Performing Arts Vienna approved the procedures of this study (reference EK Nr: 05/2020). The participants provided their written informed consent to participate in this study.

## Author contributions

SD made a substantial contribution to data acquisition, analysis, and interpretation and drafted the article. LB and WG contributed to the analysis and interpretation of the data collected. All authors equally contributed to the conception and design of the study, critically revised the article, and approved the submitted version.

## Funding

This research was supported by the Austrian Science Fund, project P32642, and the University of Oslo and the Research Council of Norway through its Centres of Excellence scheme, project number 262762.

## Conflict of interest

The authors declare that the research was conducted in the absence of any commercial or financial relationships that could be construed as a potential conflict of interest.

## Publisher's note

All claims expressed in this article are solely those of the authors and do not necessarily represent those of their affiliated organizations, or those of the publisher, the editors and the reviewers. Any product that may be evaluated in this article, or claim that may be made by its manufacturer, is not guaranteed or endorsed by the publisher.

## Footnotes

1 This is a low-pass filter used for smoothing data based on local least-squares polynomial approximation (Savitzky and Golay, 1964).

2 http://www.ableton.com

3 https://www.audacityteam.org/

4 https://www.qualisys.com/

5 https://www.openshot.org/

6 https://gorilla.sc/
